# Robust Model Predictive Control with a Dynamic Look-Ahead Re-Entry Strategy for Trajectory Tracking of Differential-Drive Robots

**DOI:** 10.3390/s26020520

**Published:** 2026-01-13

**Authors:** Diego Guffanti, Moisés Filiberto Mora Murillo, Santiago Bustamante Sanchez, Javier Oswaldo Obregón Gutiérrez, Marco Alejandro Hinojosa, Alberto Brunete, Miguel Hernando, David Álvarez

**Affiliations:** 1 Centro de Investigación en Mecatrónica y Sistemas Interactivos—MIST, Universidad Indoamérica, Av. Machala y Sabanilla, Quito 170103, Ecuador; 2 Departamento de Mecánica y Ciencias Exactas, Instituto Superior Universitario Japón, Santo Domingo 230102, Ecuador; mmora@itsjapon.edu.ec; 3 Carrera de Electricidad, Instituto Superior Tecnológico Tsa’chila, Santo Domingo 230109, Ecuador; 4 Carrera de Tecnología Superior en Mecánica Industrial, Instituto Superior Tecnológico Tsa’chila, Santo Domingo 230109, Ecuadormarcohinojosa@tsachila.edu.ec (M.A.H.); 5 Carrera de Electrónica, Instituto Superior Tecnológico Tsa’chila, Santo Domingo 230109, Ecuador; javierobregon@tsachila.edu.ec; 6 Universidad Laica Eloy Alfaro de Manabí sede Santo Domingo, Santo Domingo 230108, Ecuador; 7 Centre for Automation and Robotics (CAR UPM-CSIC), Universidad Politécnica de Madrid, 28012 Madrid, Spain; alberto.brunete@upm.es (A.B.); miguel.hernando@upm.es (M.H.); david.asanchez@upm.es (D.Á.)

**Keywords:** Model Predictive Control (MPC), differential-drive robot, trajectory tracking, re-entry strategy, robust navigation

## Abstract

Accurate trajectory tracking remains a central challenge in differential-drive mobile robots (DDMRs), particularly when operating under real-world conditions. Model Predictive Control (MPC) provides a powerful framework for this task, but its performance degrades when the robot deviates significantly from the nominal path. To address this limitation, robust recovery mechanisms are required to ensure stable and precise tracking. This work presents an experimental validation of an MPC controller applied to a four-wheel DDMR, whose odometry is corrected by a SLAM algorithm running in ROS 2. The MPC is formulated as a quadratic program with state and input constraints on linear (*v*) and angular (ω) velocities, using a prediction horizon of $N_{p}=15$ future states, adjusted to the computational resources of the onboard computer. A novel dynamic look-ahead re-entry strategy is proposed, which activates when the robot exits a predefined lateral error band (δ=0.05 m) and interpolates a smooth reconnection trajectory based on a forward look-ahead point, ensuring gradual convergence and avoiding abrupt re-entry actions. Accuracy was evaluated through lateral and heading errors measured via geometric projection onto the nominal path, ensuring fair comparison. From these errors, RMSE, MAE, $P_{95}$, and in-band percentage were computed as quantitative metrics. The framework was tested on real hardware at 50 Hz through 5 nominal experiments and 3 perturbed experiments. Perturbations consisted of externally imposed velocity commands at specific points along the path, while configuration parameters were systematically varied across trials, including the weight *R*, smoothing distance $L_{\mathrm{smooth}}$, and activation of the re-entry strategy. In nominal conditions, the best configuration (ID 2) achieved a lateral RMSE of 0.05 m, a heading RMSE of 0.06 rad, and maintained 68.8% of the trajectory within the validation band. Under perturbations, the proposed strategy substantially improved robustness. For instance, in experiment ID 6 the robot sustained a lateral RMSE of 0.12 m and preserved 51.4% in-band, outperforming MPC without re-entry, which suffered from larger deviations and slower recoveries. The results confirm that integrating MPC with the proposed re-entry strategy enhances both accuracy and robustness in DDMR trajectory tracking. By combining predictive control with a spatially grounded recovery mechanism, the approach ensures consistent performance in challenging scenarios, underscoring its relevance for reliable mobile robot navigation in uncertain environments.

## 1. Introduction

Trajectory tracking for differential drive mobile robots (DDMRs) remains a central problem in robotics, especially in applications that require high precision, robustness to disturbances, and real-world deployment. Model Predictive Control (MPC) has become a popular method for this task because it allows explicit consideration of constraints, prediction of future system behavior, and optimization over a finite horizon. Although MPC has been widely studied in simulation, its performance in real hardware under perturbations and large deviations is less frequently evaluated.

Recent works have addressed various aspects of MPC in mobile robot control. Xu et al. [1] propose a Dynamic Model Predictive Control (DMPC) for a three-wheeled independent drive and steering mobile robot (3WID3WIS), demonstrating improved lateral and heading accuracy compared to traditional MPC, especially when the curvature of the path changes. Ali, Shen and Hashim [2] introduce a safety-critical MPC with control barrier functions for differential-drive robots, focusing on obstacle avoidance and guaranteeing safety constraints alongside trajectory following. Abed et al. [3] present a nonlinear neural network fractional trajectory tracking controller for DDMRs, improving accuracy in the presence of model uncertainties. Pang et al. [4] develop a time-varying linear MPC for autonomous vehicles (which share similar challenges of lateral error and heading control), achieving stability and high precision in real road trajectories. Zhang et al. [5] survey learning-based MPC methods for path tracking control under uncertain disturbances, underlining that many methods struggle when the robot deviates significantly from the nominal path. The work by Bouzoualegh, Guechi, and Kelaiaia [6] examines the use of dynamic vs. kinematic models for MPC in DDMRs, showing that incorporating more realistic dynamics can yield better tracking but also increases susceptibility to disturbances when not properly managed.

A recurring issue in these works is that, although MPC can correct for small errors, when deviations are large (due to disturbances, external forces, slippage, or sensor errors), MPC alone may require long correction times or may lead to overshoots. This suggests the potential benefit of an auxiliary strategy that assists MPC in re-entry or realigning to the reference trajectory more aggressively or smoothly whenever the error passes a certain threshold. This re-entry strategy can reduce the deviation in the worst-case scenario, limit the time outside acceptable error bounds, and improve the robustness of the real world.

In this paper, we propose an MPC implementation in a differential drive robot augmented with a re-entry strategy that is triggered when the lateral error exceeds a fixed threshold (0.05 m). We present an extensive experimental study (8 real experiments) that compares the conditions with and without the re-entry strategy, both under normal navigation and under perturbations. Our main contributions can be summarized as follows:

- A sensor-based implementation of a Model Predictive Controller (MPC) for trajectory tracking in a four-wheel differential-drive mobile robot (DDMR), validated on real hardware under ROS 2 using SLAM-based odometry (LiDAR/IMU/encoders) for continuous state estimation.
- A novel re-entry strategy that is activated based on real-time sensory monitoring of the lateral deviation of the robot from the path. When the deviation exceeds a predefined band (δ=0.05 m), the method generates a smooth, sensor-triggered reconnection trajectory.
- Experimental evidence showing that the proposed sensing-driven control strategy enhances robustness against perturbations, reducing both lateral and heading errors, and increasing the percentage of the trajectory tracked within the validation band compared to MPC alone.
- A real-time feasibility study of the sensing-control loop, including a computational cost evaluation of the MPC pipeline with and without the re-entry mechanism. This analysis confirms that the proposed sensor-driven controller satisfies the timing constraints of the 20 ms sampling period in all tested conditions.

The rest of this paper is structured as follows. Section 2 reviews the state of the art in MPC for mobile robots. Section 3 describes the experimental DDMR used in this study, the MPC formulation, and the re-entry strategy. Section 5 discusses the implications, limitations, and proposes future research. Finally, Section 6 concludes this study.

## 2. State-of-the-Art

Model Predictive Control (MPC) is a well–established approach for trajectory tracking in wheeled mobile robots due to its ability to optimize performance under state and input constraints. Early and baseline formulations for DDMRs demonstrated the feasibility of MPC with both kinematic and dynamic models, laying the foundation for more robust and safety-aware extensions [6]. Beyond these foundations, a large body of work has explored MPC variants tailored to mobile platforms, including formulation choices (linear vs. nonlinear MPC), horizon design, and real-time implementation.

Safety-aware MPC has been advanced by incorporating Control Barrier Functions (CBFs) to enforce collision avoidance and state-space safety sets while preserving receding-horizon optimality. For DDMRs and other ground vehicles, linear or nonlinear MPC combined with barrier or feasibility constraints has been shown to maintain constraint satisfaction and stability in dynamic environments [2,7,8]. These formulations primarily target safety, feasibility, and constraint handling, but in general rely on nominal tracking when deviations occur, rather than explicitly modifying the reference to shorten recovery time.

Another active direction is robust MPC for mobile robots, where uncertainty and disturbances are handled via tube-based or dual-loop designs. Robust tube MPC, disturbance observers, and slip-aware controllers have been used in ground vehicles, skid-steer or manipulator platforms to attenuate disturbances and improve worst-case tracking guaranties [9,10,11]. Stochastic MPC has also been applied to wheeled robots to probabilistically satisfy constraints under additive disturbances [12]. Although these methods improve resilience, they still treat large deviations as disturbances around a fixed path, without a specific reincorporation mechanism designed to reshape the reference when the robot drifts beyond an application-relevant band.

There is also a growing emphasis on learning-aided or model-enhanced MPC to improve prediction fidelity and tracking performance by combining data-driven models (e.g., Gaussian processes) with predictive control to mitigate modeling mismatch under uncertain conditions [5,13]. Similar ideas are explored for non-holonomic and skid-steer robots, where NMPC, observers, or slip compensation are used to maintain precision under wheel–ground interactions [14]. These approaches typically improve the average tracking accuracy, but practical recovery from significant deviations (e.g., slippage, pushes, or sensing failures) still depends on the ability of the MPC to quickly converge again.

In addition, recent research explores hybrid frameworks where MPC is combined with dynamic trajectory re-planning or online path updating, enabling the robot to modify the planned path in real time under changing environments or moving obstacles. Examples include MPC-based re-planning under uncertainty for manipulators [15], MPC combined with neurodynamic optimization for dynamic obstacle avoidance in car-like robots [16], and MPC-based path planning in multi-robot environments [17]. These works underline that MPC-based dynamic replanning remains an active line of research for mobile platforms operating in dynamic or uncertain conditions, supporting the motivation for lightweight replanning or re-entry mechanisms like the one presented in this study.

Time-varying or spatial MPC formulations have also been proposed to handle curvature changes and non-holonomic constraints on mobile platforms [14]. These contributions improve nominal tracking and robustness. However, most lack an evaluation protocol centered on band occupancy (time in a narrow lateral error band).

In summary, the previous state of the art demonstrates (i) safety-aware MPC with CBFs, (ii) robust/tube or stochastic MPC for bounded uncertainties, and (iii) learning-augmented MPC for better prediction. What remains under-reported is an experimentally validated practical mechanism to assist MPC during significant deviations by temporarily reshaping the reference trajectory. Our work addresses this gap through a smooth re-entry strategy activated by a threshold and evaluated using application-oriented metrics such as time within a narrow lateral band, number of band exits, and number of re-entries, validated in real-robot experiments under both nominal operation and deliberate perturbations.

## 3. Methods

### 3.1. Robot Platform

The experimental platform is a four-wheel differential-drive robot assembled on a Recon Chassis Kit (ServoCity, Winfield, KS, USA). Each wheel is actuated by a DC motor of type 5203 series Yellow Jacket Planetary Gear Motor (goBILDA, Winfield, IL, USA), with a 19.2:1 reduction ratio and a nominal speed of 312 RPM. Each motor integrates a quadrature encoder that provides a resolution of 537.7 pulses per revolution (PPR) in the output shaft. The wheels are filled with foam, ensuring stable operation on rigid outdoor surfaces. The actuation system is driven by two dual motor drivers (RoboClaw 2x15A), one per side of the differential configuration. These drivers are commanded by an MCU (Nucleo STM32F207ZG) that receives velocity commands and translates them into pulse-per-second signals for the motors. The robot is also equipped with a WitMotion WT901C-TTL 9-axis IMU (accelerometer, gyroscope, magnetometer, and inclinometer) and a Slamtec A2 2D LiDAR with a maximum range of 16 m, used for SLAM and localization.

The SLAM module is responsible for fusing wheel encoder odometry, IMU data, and 2D LiDAR scans to build the map and estimate the robot pose for trajectory tracking. We use the slam_toolbox package from ROS2 in online asynchronous mapping mode, which provides the corrected odometry through the /odom_filtered topic. The raw odometry is integrated in the /base_footprint to /odom transform, while LiDAR scan matching refines the /map to /odom transform, producing the SLAM-based pose feedback used by the MPC.

The reference trajectory used in all experiments consists of 1859 spatial poses (x,y,θ) recorded from the SLAM-based odometry. Its total length is approximately 20 m and consecutive points are sampled with an average spacing of 1.1 cm (maximum around 1.5 cm). The path includes straight segments and curved turns, and is used as the nominal trajectory P throughout the study.

The system is powered by a Tattu G-tech 5200 mAh 4S 35C 14.8V LiPo battery. The onboard computer is an Intel NUC BNUC11TNKV70002 11 Pro, equipped with an Intel Core i7-1185G7 processor (4 cores, 8 threads, base frequency 3.0 GHz, turbo up to 4.8 GHz, 12 MB cache), 16 GB RAM, and a 500 GB SSD, powered by a Tattu G-tech 5200 mAh 4S 35C 14.8V LiPo battery. This configuration provides sufficient computational power to reliably execute the control loop at 50 Hz (sampling time $T_{s}=20$ ms), while also handling the perception, state estimation, and communication tasks required by the robot.

**Figure 1:**
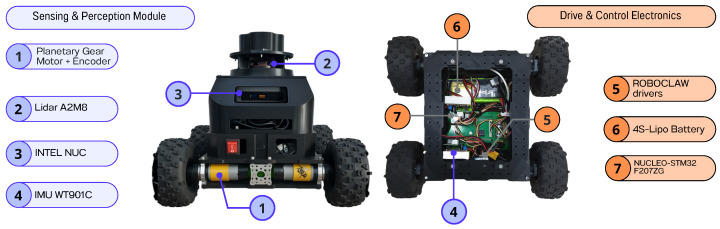
Hardware architecture of the mobile robot, showing the two main subsystems: (**left**) the Sensing and Perception Module including the LiDAR, planetary geared motors with encoders, IMU and on-board computer; (**right**) the Drive and Control Electronics including the motor drivers, embedded controller and power supply.

The control architecture is implemented in ROS 2 Foxy Fitzroy on Ubuntu 20.04 (Focal). The slam_toolbox package was used to perform mapping and localization, combining odometry from wheel encoders, IMU, and LiDAR scans. This fusion provided an accurate estimate of the odometry suitable for feedback to the MPC implemented in this study. Figure 1 shows the hardware layout of the experimental robot, highlighting the perception module and the embedded control electronics.

The experiments were carried out on a flat concrete surface outside, surrounded by buildings that improved the accuracy of the location of the SLAM system. The physical platform is capable of reaching linear velocities of up to approximately 1.7 m/s and angular velocities close to 2.0 rad/s. However, operating near these limits can place excessive stress on the motors and reduce control robustness. For this reason, this study was configured with conservative bounds of v=±1.5 m/s and ω=±1.75 rad/s. These limits were selected as a trade-off between exploiting the full capability of the robot and ensuring safe and reliable operation during experiments.

**Figure 2:**
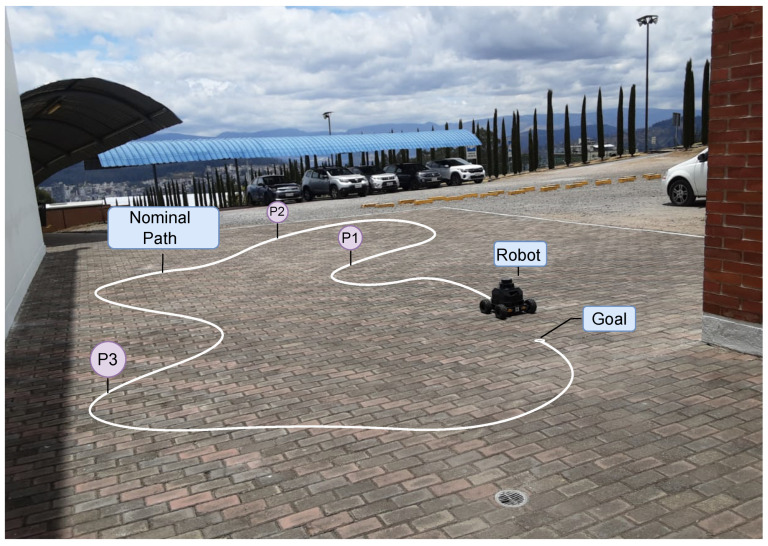
Experimental robot platform and nominal path with labeled perturbation points (P1–P3) used for subsequent evaluation.

The experimental setup is illustrated in Figure 2. The robot follows a predefined reference trajectory on a tiled floor, while three perturbation points (P1–P3) are marked along the path. These points are later used to inject controlled disturbances into the system, allowing us to evaluate the robustness of the control strategy under non-ideal conditions.

### 3.2. MPC Formulation

The trajectory tracking problem is formulated as a discrete-time Model Predictive Control (MPC) optimization. The robot state vector is

(1) $$x_{k}={\left[\begin{array}{ccc} x_{k} & y_{k} & \theta_{k} \end{array}\right]}^{⊤},$$

where $\left(x_{k},y_{k}\right)$ are the Cartesian coordinates and $\theta_{k}$ is the heading angle at time step *k*. The control inputs are the commanded linear and angular velocities

(2) $$u_{k}={\left[\begin{array}{cc} v_{k} & \omega_{k} \end{array}\right]}^{⊤}.$$

Let the robot state at time *k* be $x_{k}={\left[x_{k},\;y_{k},\;\theta_{k}\right]}^{⊤}$, whose positional component $p_{k}={\left[x_{k},\;y_{k}\right]}^{⊤}$ is used for geometric computations on the reference path P. The nominal path is defined as a finite sequence of *M* spatial poses,

(3) $$P={\left\{\left(x_{i},y_{i},\theta_{i}\right)\right\}}_{i=1}^{M},$$

where the positional component $p_{i}={\left[x_{i},\;y_{i}\right]}^{⊤}$ denotes the Cartesian coordinates and $\theta_{i}$ the heading of the *i*-th spatial point.

At each control cycle, the algorithm computes the lateral tracking error $e_{y}\left(k\right)$ between the robot position and each point of P as

(4) $$e_{y}\left(k\right)={\left(p_{k}-p_{i}\right)}^{⊤}\left[\begin{array}{c} -\sin\theta_{i}\\ \cos\theta_{i} \end{array}\right],$$

and determines the closest point on the path as

(5) $$p_{c}=\arg\min_{p_{i}\in P}\left|e_{y}\left(k\right)\right|.$$

Consequently, the pose $p_{c}=\left(x_{c},\;y_{c},\;\theta_{c}\right)$ defines the local tangent direction $t_{c}={\left[\cos\theta_{c},\;\sin\theta_{c}\right]}^{⊤}$ and serves as the spatial anchor for the prediction horizon. The scalar $e_{y}\left(k\right)$ quantifies the signed perpendicular deviation of the robot from the nominal path and is also used as the activation criterion for the re-entry mechanism (Section 3.3).

The MPC then extracts from P a local reference horizon $H_{k}$ of length $N_{p}$, composed of the sequence of poses ahead of $p_{c}$:

(6) $$H_{k}=\left\{x_{c+1}^{ref},\;x_{c+2}^{ref},\;\ldots ,\;x_{c+N_{p}}^{ref}\right\},$$

where each $x_{c+i}^{ref}={\left[x_{c+i},\;y_{c+i},\;\theta_{c+i}\right]}^{⊤},i=1,\ldots ,N_{p},$ represents a spatial reference point along the path. Each element in this horizon corresponds to a discrete prediction step associated with the controller sampling period $T_{s}$, so that the predicted state $x_{k+i}$ represents the expected robot pose at time $t_{k}+iT_{s}$. This spatial re-alignment ensures that the reference horizon always begins from the point on P closest to the robot, providing consistent geometric tracking even when the path was recorded at variable speeds. If $|e_{y}\left(k\right)|>\delta$, the nominal horizon $H_{k}$ is replaced by a re-entry horizon as described in Section 3.3.

The nonlinear kinematic model of the differential-drive robot is

(7) $$\begin{array}{cc} x_{k+1} & =x_{k}+T_{s}\;v_{k}\cos\left(\theta_{k}\right), \end{array}$$

(8) $$\begin{array}{cc} y_{k+1} & =y_{k}+T_{s}\;v_{k}\sin\left(\theta_{k}\right), \end{array}$$

(9) $$\begin{array}{cc} \theta_{k+1} & =\theta_{k}+T_{s}\;\omega_{k}, \end{array}$$

where $T_{s}$ is the sampling time. For prediction within MPC, the model is linearized at each step around the operating point $\left({\hat{x}}_{k},{\hat{y}}_{k},{\hat{\theta }}_{k},{\hat{v}}_{k},{\hat{\omega }}_{k}\right)$, yielding

(10) $$x_{k+1}\approx A_{k}x_{k}+B_{k}u_{k},$$

where $A_{k}\in R^{3\times 3}$ and $B_{k}\in R^{3\times 2}$ are Jacobians.

Given a prediction horizon of length $N_{p}$, the stacked predicted state and control vectors are defined as

(11) $$X={\left[\begin{array}{ccc} x_{k+1}^{⊤} & \ldots & x_{k+N_{p}}^{⊤} \end{array}\right]}^{⊤},\;U={\left[\begin{array}{cccc} u_{k}^{⊤} & u_{k+1}^{⊤} & \ldots & u_{k+N_{p}-1}^{⊤} \end{array}\right]}^{⊤}.$$

Here, $X\in R^{3N_{p}}$ contains the predicted robot states, while $U\in R^{2N_{p}}$ represents the sequence of control inputs to be optimized by the MPC. Since no reference control sequence is provided by the nominal path, the control inputs are computed online as decision variables of the optimization problem.

The cost function penalizes both the deviation from the spatially aligned reference trajectory and the magnitude of the control effort, according to

(12) $$J=\sum_{i=1}^{N_{p}}\left(∥x_{k+i}-x_{c+i}^{ref}{∥}_{Q}^{2}+{∥u_{k+i-1}∥}_{R}^{2}\right),$$

where $Q\in R^{3\times 3}⪰0$ and $R\in R^{2\times 2}≻0$ are diagonal weighting matrices for the state and input, respectively. The matrix *Q* shapes the relative importance of position and orientation errors in the tracking term, while *R* penalizes excessive control effort and indirectly influences the smoothness of the commanded velocities. The vectors $x_{c+i}^{ref}$ are the $N_{p}$ reference poses contained in $H_{k}$, and thus define the local tracking targets used in the optimization. This cost can be written in quadratic form as

(13) $$J=\frac{1}{2}U^{⊤}HU+f^{⊤}U,$$

where

(14) $$H=\Gamma^{⊤}Q_{b}\Gamma +R_{b},\;f=\Gamma^{⊤}Q_{b}\left(\Phi x_{k}-X^{ref}\right),$$

with Φ and Γ the prediction matrices. The matrix $\Phi \in R^{3N_{p}\times 3}$ propagates the current state through the prediction horizon, while $\Gamma \in R^{3N_{p}\times 2N_{p}}$ is the control-to-state matrix (Toeplitz form) that maps the control input sequence to the predicted state trajectory:

(15) $$X=\Phi x_{k}+\Gamma U.$$

The block-diagonal matrices $Q_{b}=\mathrm{blkdiag}\left(Q,\ldots ,Q\right)$ and $R_{b}=\mathrm{blkdiag}\left(R,\ldots ,R\right)$ encode the stage weights along the horizon.

The MPC optimization problem at each sampling instant is therefore

(16) $$\begin{array}{cc} \min_{U\in R^{2N_{p}}}\; & J \end{array}$$

(17) $$\begin{array}{cc} s.t.\; & x_{k+i+1}=A_{k}x_{k+i}+B_{k}u_{k+i}, \end{array}$$

(18) $$\begin{array}{cc} \; & v_{\min}\leq v_{k+i}\leq v_{\max}, \end{array}$$

(19) $$\begin{array}{cc} \; & \omega_{\min}\leq \omega_{k+i}\leq \omega_{\max}, \end{array}$$

(20) $$\begin{array}{cc} \; & \forall i=0,\ldots ,N_{p}-1. \end{array}$$

Although the optimization is carried out over the control sequence U, the cost function J(U) penalizes the deviation between the predicted and reference trajectories, thus driving the robot state toward the desired path while regulating the control effort. The linear velocity was constrained to $v_{\min}=-1.5$ m/s and $v_{\max}=+1.5$ m/s, and the angular velocity to $\omega_{\min}=-1.75$ rad/s and $\omega_{\max}=+1.75$ rad/s, reflecting the physical capabilities of the drive motors. Although no additional constraints on the rate of change of *v* or ω were imposed, the weighting on control effort smooths the commanded inputs, preventing abrupt variations within the feasible bounds.

At each step, only the first optimal input $u_{k}^{★}={\left[v_{k}^{★},\omega_{k}^{★}\right]}^{⊤}$ is applied to the robot (receding horizon principle). The complete online implementation is summarized in Algorithm 1, which directly reflects the Python 3.8 node executed in the robot; it reads odometry, constructs the prediction horizon, builds the quadratic program, solves the optimization, and publishes the command. Optional disturbances can be enabled to test robustness. Algorithm 1 calls Algorithm 2 to compute the reference horizon $H_{k}$ (nominal or re-entry) at each iteration.
**Algorithm 1 **Online MPC Control Loop with Optional Disturbances and Re-Entry StrategyRequire: $T_{s}$, $N_{p}$, Q⪰0, R≻0, bounds $u_{\min},u_{\max}$, band δ, reference path P, optional disturbances D1:**while** goal not reached **do**2:   Read $x_{k}={\left[x_{k},y_{k},\theta_{k}\right]}^{⊤}$3:   Compute lateral errors $e_{y}\left(k\right)$ for all $p_{i}\in P$4:   Find closest point $p_{c}=\left(x_{c},y_{c},\theta_{c}\right)=\arg\min_{p_{i}\in P}\left|e_{y}\left(k\right)\right|$
5:   $H_{k}$ ← ComputeReferenceHorizon$\left(x_{k},p_{c},P,N_{p},\delta \right)$▹ see Algorithm 26:   Build $X^{ref}$ by stacking $x_{c+i}^{ref}$ from $H_{k}$7:   Linearize model at $\left({\hat{x}}_{k},{\hat{y}}_{k},{\hat{\theta }}_{k},{\hat{v}}_{k},{\hat{\omega }}_{k}\right)$ to obtain $A_{k},B_{k}$8:   Construct prediction matrices (Φ,Γ) from $\left(A_{k},B_{k}\right)$ and $N_{p}$9:   Form $Q_{b}=\mathrm{blkdiag}\left(Q,\ldots ,Q\right)$, $R_{b}=\mathrm{blkdiag}\left(R,\ldots ,R\right)$10:   Compute $H=\Gamma^{⊤}Q_{b}\Gamma +R_{b}$,   $f=\Gamma^{⊤}Q_{b}\left(\Phi x_{k}-X^{ref}\right)$11:   Solve $\min_{U\in R^{2N_{p}}}\frac{1}{2}U^{⊤}HU+f^{⊤}U$ s.t. $u_{\min}\leq U\leq u_{\max}$12:   Extract and apply $u_{k}^{★}={\left[v_{k}^{★},\omega_{k}^{★}\right]}^{⊤}$13:   **if** a disturbance $(p_{j},v_{j},\omega_{j},\tau_{j})\in D$ is due **then**14:      Apply $\left(v_{j},\omega_{j}\right)$ for $\tau_{j}$ seconds; mark as applied; **continue**15:   Update metrics ($e_{y}$, band exits, # re-entries)

### 3.3. Re-Entry Strategy

Although MPC can correct small deviations, large disturbances or wheel slip can drive the robot outside the admissible lateral band δ=0.05 m. In such cases, tracking the nominal path directly would require aggressive maneuvers, leading to instability or loss of robustness. To mitigate this, a *re-entry strategy* is introduced to temporarily replace the nominal reference horizon with a smooth interpolated sequence that guides the robot back into the band.

In each control cycle, the closest point $p_{c}=\left(x_{c},\;y_{c},\;\theta_{c}\right)\in P$ is already calculated as part of the horizon construction described in Section 3.2 and detailed in Algorithm 1. When the signed lateral deviation satisfies $|e_{y}\left(k\right)|>\delta$, the re-entry mechanism is activated.

A smoothing point $p_{s}=\left(x_{s},\;y_{s},\;\theta_{s}\right)\in P$ is selected ahead of $p_{c}$ at a prescribed look-ahead distance $L_{\mathrm{smooth}}$ such that the Euclidean distance between them satisfies

(21) $$∥p_{s}-p_{c}∥\geq L_{\mathrm{smooth}}.$$

The re-entry seed is then built as

(22) $$S=\{p_{r},\;p_{s},\;x_{s+1}^{ref},\ldots ,x_{s+N_{p}-2}^{ref}\},$$

where $p_{r}={\left[x_{k},\;y_{k},\;\theta_{r}\right]}^{⊤}$ represents the robot pose with an adjusted heading $\theta_{r}$ oriented toward $p_{s}$, defined as

(23) $$\theta_{r}=\tan^{-1}\;\left(\frac{y_{s}-y_{k}}{x_{s}-x_{k}}\right),$$

This alignment ensures that the first reference orientation in the temporary horizon is geometrically consistent with the direction of re-entry, rather than maintaining the robot’s current heading $\theta_{k}$.

Next, the seed sequence S is interpolated to obtain continuous functions x(s), y(s), and θ(s) parameterized by arc-length *s*. The cumulative distance along S is computed as

(24) $$s_{j}=s_{j-1}+∥{\left[x_{j},\;y_{j}\right]}^{⊤}-{\left[x_{j-1},\;y_{j-1}\right]}^{⊤}∥,\;s_{0}=0,$$

and resampled uniformly at arc-length increments Δs:

(25) $$s_{\ell }=\ell \cdot \Delta s,\;\ell =0,1,\ldots ,\left\lfloor s_{\max}/\Delta s\right\rfloor .$$

Evaluating the interpolants at $\left\{s_{\ell }\right\}$ yields

(26) $$\tilde{P}=\left\{\left(x\left(s_{\ell }\right),\;y\left(s_{\ell }\right),\;\theta \left(s_{\ell }\right)\right)\right\},$$

from which the first $N_{p}$ samples are taken as the re-entry horizon $H_{k}$. If fewer than $N_{p}$ samples are available (e.g., near the end of the path), the horizon is padded with copies of the last nominal pose to maintain constant length.

**Figure 3:**
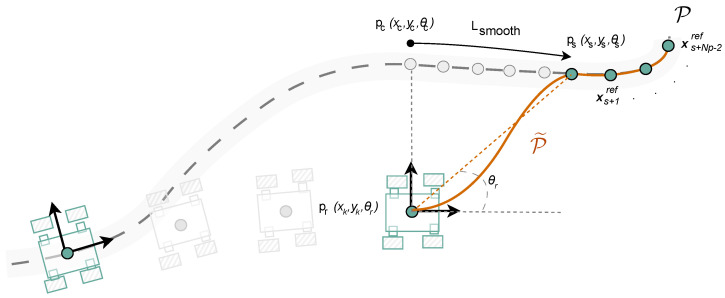
Re-entry strategy: dynamic look-ahead smoothing horizon constructed when the robot exits the nominal path. The dashed gray line represents the nominal reference path, while the solid orange line denotes the re-entry trajectory. Arrows show the robot orientation and motion direction, and colored points correspond to the robot pose and reference points used by the MPC.

This re-entry mechanism is depicted in Figure 3. Starting from the robot pose $p_{r}$, the algorithm selects a smoothing point $p_{s}$ ahead of the nearest nominal point $p_{c}$ at a prescribed look-ahead distance $L_{\mathrm{smooth}}$. A new interpolated trajectory $\tilde{P}$ is then generated by combining $p_{r}$, $p_{s}$, and the following $N_{p}-2$ nominal points. This interpolated sequence defines the temporary horizon for the MPC, ensuring smooth convergence back to the reference path P.

When $|e_{y}|\leq \delta$, the horizon $H_{k}$ coincides with the next $N_{p}$ nominal points $\left\{x_{c+1}^{ref},\ldots ,x_{c+N_{p}}^{ref}\right\}$. Otherwise, $H_{k}$ is replaced by the resampled re-entry horizon obtained from $\tilde{P}$. In both cases, $H_{k}$ is stacked into $X^{ref}$ for the quadratic program. The complete implementation, consistent with the Python node executed on the robot, is summarized in Algorithm 2.
**Algorithm 2 **ComputeReferenceHorizon: Nominal or Re-Entry (arc-length resampling)**Require:** State $x_{k}={\left[x_{k},y_{k},\theta_{k}\right]}^{⊤}$, nominal path $P={\left\{\left(x_{i},y_{i},\theta_{i}\right)\right\}}_{i=1}^{M}$, horizon $N_{p}$, band δ=0.05m, look-ahead $L_{\mathrm{smooth}}$1:Find closest point $p_{c}=\left(x_{c},y_{c},\theta_{c}\right)\in P$ to $\left(x_{k},y_{k}\right)$; compute $e_{y}$2:**if** $|e_{y}|\;\leq \delta$**then**▹ Inside band3:    $H_{k}\leftarrow \left\{x_{c+1}^{ref},x_{c+2}^{ref},\ldots ,x_{c+N_{p}}^{ref}\right\}$4:    **if** $|H_{k}|\;<N_{p}$ **then**
5:        Pad with $x_{M}^{ref}$ until $|H_{k}|\;=N_{p}$6:    **return** $H_{k}$▹ Outside band: re-entry7:Select smoothing point $p_{s}=\left(x_{s},y_{s},\theta_{s}\right)\in P$ such that $∥p_{s}-p_{c}∥\;\geq L_{\mathrm{smooth}}$8:Define first point (robot): $p_{r}=\left(x_{k},y_{k},\theta_{r}\right)$ with $\theta_{r}=\tan^{-1}\;\left(\frac{y_{s}-y_{k}}{x_{s}-x_{k}}\right)$9:Build seed $S=\{p_{r},p_{s},x_{s+1}^{ref},\ldots ,x_{s+N_{p}-2}^{ref}\}$10:Compute cumulative arc-length $s_{0}=0$, $s_{j}=s_{j-1}+∥\left[x_{j},y_{j}\right]-\left[x_{j-1},y_{j-1}\right]∥$11:Fit interpolants x(s),y(s),θ(s) (cubic if |S|≥4, else linear)12:Resample at uniform arc steps: $s_{\ell }=\ell \cdot \Delta s,\;\ell =0,\ldots ,L/\Delta s$13:Define $H_{k}={\left\{\left(x\left(s_{\ell }\right),y\left(s_{\ell }\right),\theta \left(s_{\ell }\right)\right)\right\}}_{\ell =1}^{N_{p}}$14:**if** $|H_{k}|\;<N_{p}$ 
**then**15:      Pad with $x_{M}^{ref}$ until $|H_{k}|\;=N_{p}$16:**return** $H_{k}$

### 3.4. Stability Considerations

The proposed MPC controller adopts the standard quadratic stage cost in tracking error and control effort and relies on the discretized linearized model $x_{k+1}=Ax_{k}+Bu_{k}$, where the pair (A,B) is stabilizable. This means that the control inputs $u={\left[v,w\right]}^{⊤}$ are sufficient to influence all potentially unstable modes of the system so that stabilizing feedback can be designed. In this context, the MPC cost can be interpreted as an energy-like Lyapunov function, since it measures how far the robot is from the desired trajectory. When the robot approaches the reference, this energy decreases. More precisely, if $V\left(x_{k}\right)=J\left(x_{k}\right)$ denotes the optimal cost at time *k*, the MPC update satisfies the Lyapunov decrease condition $V\left(x_{k+1}\right)-V\left(x_{k}\right)<0$, meaning that this energy-like quantity strictly decreases along the closed-loop trajectory. This is the classical argument used in MPC theory to guarantee closed-loop stability even without terminal constraints or terminal costs. For differential-drive robots, Worthmann et al. [18] formally proved closed-loop stability using MPC without terminal stabilizing ingredients, which directly supports our formulation. Furthermore, the general theoretical foundations of stability for MPC schemes based solely on the stage cost are rigorously established in the nonlinear MPC framework of Grüne and Pannek [19], reinforcing the validity of the control strategy adopted in this work. Note also that the re-entry trajectory is only used as a bounded, smooth and feasible external reference signal and does not modify the MPC formulation; therefore, the stability guarantees of the baseline controller remain preserved.

### 3.5. Experimental Setup and Evaluation

To validate the proposed MPC controller and the re-entry strategy, eight experiments were carried out on the real differential-drive robot. For clarity, experiments are renumbered consecutively from 1 to 8 in this paper. All runs used a fixed lateral band of δ=0.05 m and a prediction horizon of $N_{p}=15$, corresponding to a prediction interval of 0.3 s given the controller sampling period $T_{s}=0.02$ s. This horizon length was selected as a compromise between computational feasibility and sufficient anticipation of the robot dynamics, since larger horizons significantly increased the solver time on the onboard computer.

The diagonal weighting matrix Q=[25.0,25.0,20.0] was kept fixed across all experiments so that the relative importance of position (x,y) and heading (θ) errors remained constant. These values were empirically chosen to give slightly higher priority to position accuracy than to orientation correction, promoting smooth yet precise trajectory tracking. Fixing *Q* allows us to evaluate the effect of different configurations of *R* on the trade-off between tracking accuracy and control smoothness without altering the tracking priority itself. Smaller *R* values lead to faster and more aggressive control actions, whereas larger *R* values promote smoother but slower responses. Additionally, both linear and cubic interpolation schemes were tested to assess the influence of trajectory smoothing on the re-entry behavior. Table 1 and Table 2 summarize the complete configurations. The column $L_{\mathrm{smooth}}$ indicates the look-ahead distance used to place the smoothing point when re-entry is active (Section 3.3).

The second part introduces deliberate perturbations to probe robustness. These perturbations were triggered once the robot progressed beyond predefined indices of the nominal path $P={\left\{\left(x_{i},y_{i},\theta_{i}\right)\right\}}_{i=1}^{M}$, specifically at i∈{500,1000,1500}. Each perturbation overrides the MPC output for a fixed duration with the specified velocity command:

P1: (v,ω)=(0.5m/s,−0.75rad/s) for 3.0 s, forward motion with a strong right-turn.
P2: (v,ω)=(0.5m/s,−0.5rad/s) for 2.0 s, forward motion with a moderate right turn.
P3: (v,ω)=(0.2m/s,1.0rad/s) for 2.0 s, forward motion with a left turn.

These cover sudden heading changes, brief stops/slowdowns, and lateral deviations.

As evaluation metrics, the Root Mean Square Error (RMSE), Mean Absolute Error (MAE), and the 95th percentile of the absolute lateral deviation ($P_{95}$) were computed from the lateral error at the robot position $p_{k}=\left(x_{k},y_{k}\right)$ as follows:

(27) $$\begin{array}{cc} \mathrm{RMSE} & =\sqrt{\frac{1}{N}\sum_{k=1}^{N}e_{y}{\left(k\right)}^{2}}, \end{array}$$

(28) $$\begin{array}{cc} \mathrm{MAE} & =\frac{1}{N}\sum_{k=1}^{N}\left|e_{y}\left(k\right)\right|, \end{array}$$

(29) $$\begin{array}{cc} P_{95} & =95\mathrm{th}\;\mathrm{percentile}\;\mathrm{of}\;|e_{y}\left(k\right)|, \end{array}$$

where *N* is the number of samples and $T_{s}$ is the sampling period. The portion of the trajectory in which the lateral error remains within the validation band δ=0.05 m is also quantified. This metric is computed as

(30) $${\mathrm{Pct}}_{\mathrm{InBand}}=100\cdot \frac{1}{N}\sum_{k=1}^{N}\left[\;|e_{y}\left(k\right)|\leq \delta \;\right],$$

The resulting value expresses the fraction of the trajectory contained within the ±δ band, normalized to a percentage scale.

In addition to the lateral error, a heading error $e_{\theta }\left(k\right)$ is calculated by comparing the measured orientation of the robot $\theta_{k}$ with the orientation $\theta_{c}$ of the tangent at the projection point $p_{c}$. This ensures that the heading deviation is always evaluated consistently with respect to the closest segment of the nominal path. Quantitative metrics such as RMSE, MAE and the 95th percentile are then obtained for $e_{\theta }\left(k\right)$ using the same formulas as above.

Beyond tracking accuracy, the smoothness and dynamic quality of control signals were also assessed. Specifically, the *RateRMS* and *JerkRMS* metrics quantify the root mean square of the rate of change and the second derivative (jerk) of the commanded linear and angular velocities, respectively, reflecting how abruptly the controller varies its commands. Finally, the Integrated Smoothness Number (ISN) provides a dimensionless indicator of overall control smoothness, with higher values (ISN≈1) indicating smoother and more stable action.

## 4. Results

This section presents the quantitative and qualitative evaluation of the proposed MPC controller under two sets of conditions: (i) nominal operation without external perturbations (IDs 1–5) and (ii) perturbed operation with three injected disturbances (IDs 6–8). The results are reported both in tabular form, highlighting performance metrics such as lateral and angular errors, and graphically, showing the robot trajectories, error profiles, and control signals.

### 4.1. Experiments Under Nominal Conditions

**Figure 4:**
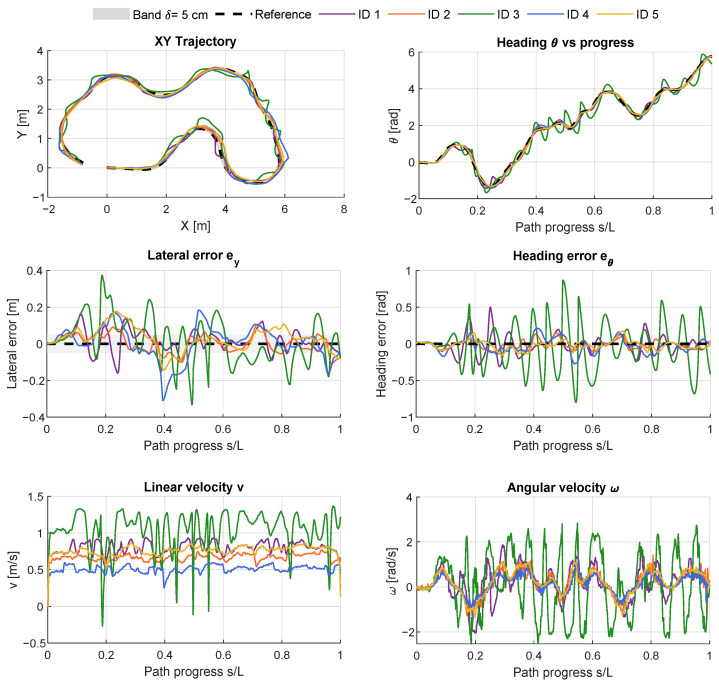
Experiments under nominal conditions (IDs 1–5) in Table 1. Comparison of the nominal path against tracked trajectories and error profiles. The *x*-axis represents the normalized path progress s/L, with *s* the arc length traveled along the reference trajectory and *L* its total length.

Table 3 summarizes the quantitative results for experiments under nominal conditions (IDs 1–5), which are consistent with the qualitative observations in Figure 4. All controllers achieved stable trajectory tracking, although with different levels of accuracy and control smoothness. The best overall performance was obtained in ID 2 (active re-entry, R=[1.0,2.0], $L_{\mathrm{smooth}}=0.25$ m), which yielded the lowest lateral RMSE (0.05 m), smallest P95 error (0.10 m), and the highest in-band operation (68.8%). In terms of control behavior, ID 2 also achieved balanced smoothness, with moderate rate and jerk values (${\mathrm{RateRMS}}_{v}=1.05$ m/s^2^, ${\mathrm{RateRMS}}_{\omega }=8.21$ rad/s^2^; ${\mathrm{JerkRMS}}_{v}=68.44$ m/s^3^, ${\mathrm{JerkRMS}}_{\omega }=661.33$ rad/s^3^) and a high smoothness index (ISN=0.95). ID 1 showed a comparable response (lateral RMSE 0.06 m, ISN=0.96), remaining smooth and well-centered along the nominal trajectory. By contrast, ID 3 (reduced weights, R=[0.5,1.0]) exhibited a more aggressive and oscillatory response, with increased lateral RMSE (0.15 m), higher ${\mathrm{RateRMS}}_{\omega }=16.88$ rad/s^2^ and ${\mathrm{JerkRMS}}_{\omega }=1310.98$ rad/s^3^, and only 25.2% of the run within the ±5 cm band. ID 4 achieved intermediate performance (lateral RMSE 0.10 m, ${\mathrm{RateRMS}}_{\omega }=8.12$ rad/s^2^), while the baseline case without re-entry (ID 5) maintained smooth velocity profiles (ISN=0.97) but showed lower robustness and slightly larger tracking deviations.

The velocity subplots in Figure 4 provide further insight. In ID 3, the smaller values of *R* produced stronger corrective actions, which appear as high-amplitude oscillations in both *v* and ω. In contrast, ID 4 (larger *R*) generated slower commands, but this did not reduce the overall error as the robot required more time to re-enter the nominal path compared to ID 2. This behavior illustrates the trade-off between control aggressiveness and re-entry speed.

### 4.2. Experiments Under Perturbations

Table 4 reports the results for IDs 6–8, where external disturbances were injected into the velocity commands.

As expected, the disturbances significantly affected the tracking accuracy of all controllers. ID 6, with the same configuration as the best nominal case (ID 2), achieved the most robust response under perturbations, with a lower lateral RMSE (0.12 m) and a higher percentage of in-band operation (51.4%) compared to IDs 7 and 8. In terms of controller behavior, ID 6 maintained moderate smoothness under disturbances (${\mathrm{RateRMS}}_{v}=2.19$ m/s^2^, ${\mathrm{RateRMS}}_{\omega }=10.34$ rad/s^2^; ${\mathrm{JerkRMS}}_{v}=146.08$ m/s^3^, ${\mathrm{JerkRMS}}_{\omega }=773.85$ rad/s^3^) and preserved a high smoothness index (ISN=0.95). By contrast, ID 7, configured with smaller *R* values, produced more aggressive but less stable control actions (higher rate and jerk values, ISN=0.94), while ID 8 (inactive re-entry) resulted in smoother commands (ISN=0.97) but prolonged deviations from the reference path and a lower in-band percentage (29.4%).

**Figure 5:**
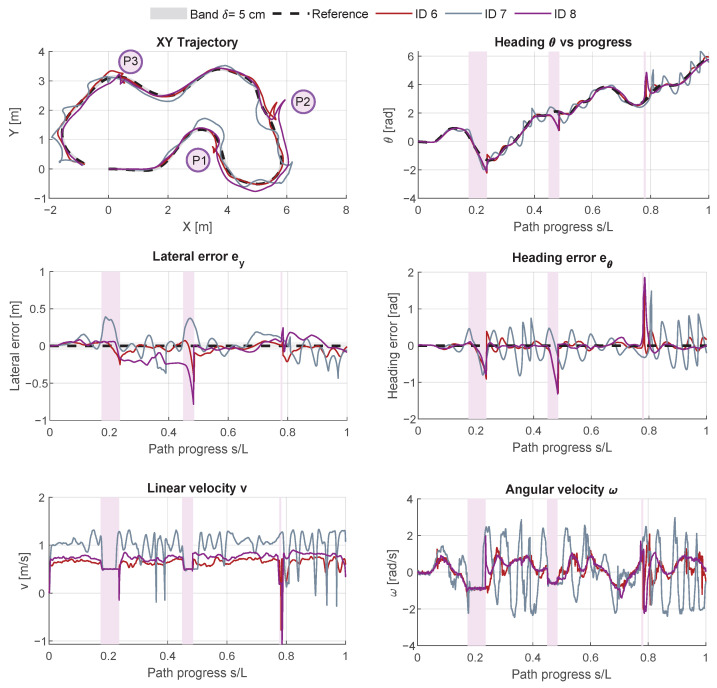
Experiments under perturbed conditions (IDs 6–8) in Table 2. Shaded regions correspond to perturbations P1–P3. The *x*-axis represents the normalized path progress s/L, with *s* the arc length traveled along the reference trajectory and *L* its total length.

Figure 5 illustrates these effects. The shaded bands (P1–P3) indicate the intervals of injected perturbations. At P1 (strong right turn while moving forward), the robot is deflected to the right; ID 6 shows a rapid return to the band, whereas IDs 7 and 8 drift further before re-aligning. At P2 (moderate right turn), the induced deviation is smaller and the recovery of ID 6 is again faster than IDs 7–8. Finally, at P3 (left turn), the largest heading excursion is observed; ID 7 reacts more aggressively with visible oscillations before stabilizing, while ID 6 maintains a smoother recovery. These behaviors are evident in both the lateral error and velocity subplots, where the shaded bands delineate disturbance and re-entry phases. It should be noted that all subplots expressed with respect to the nominal path progress (s/L)—namely lateral error, heading θ, heading error $e_{\theta }$, and the velocity profiles—are affected by this parameterization. Consequently, the shaded region at P3 appears narrower because the perturbation commands induce limited forward motion along the path, which reduces the apparent duration of the disturbance when plotted in normalized progress coordinates.

### 4.3. Sensitivity Analysis

The performance of the controller was evaluated under different parameter configurations to assess its sensitivity and to obtain practical tuning guidelines. In Table 3, the input weight *R*, the smoothing distance $L_{\mathrm{smooth}}$, the interpolation mode, and the activation of the re-entry strategy were considered. Reducing $L_{\mathrm{smooth}}$ from 0.30 m to 0.25 m decreases the lateral RMSE by approximately 15% and increases the in-band operation from 63.3% to 68.8%, indicating that $L_{\mathrm{smooth}}$ effectively trades off re-entry speed against smoothness.

The parameter *R* has a strong influence on stability. Lower penalization (R=[0.5,1.0]) increases the lateral RMSE from 0.05 m to 0.15 m (200% increase) and reduces the in-band percentage from 68.8% to 25.2% (63% reduction), while higher *R* produces smoother and less oscillatory control but slower re-entry. Under perturbations (Table 4), this trend is maintained, the RMSE increases from 0.12 m to 0.19 m (58% increase) when using a smaller *R*. These observations provide a practical tuning rule for *R*, in which larger values improve smoothness and robustness, while smaller values lead to faster but more aggressive convergence.

The type of interpolation also plays a role. Cubic interpolation yields the lowest nominal RMSE (0.05 m), compared to linear interpolation (0.06 m), suggesting a small but measurable improvement in convergence and smoothness. The activation of the re-entry mechanism also exhibits a clear sensitivity effect. Disabling the strategy (ID 5) increases the lateral RMSE to 0.07 m and reduces the in-band operation to 52.6%, while enabling it (ID 2) improves these values to 0.05 m and 68.8%. Under perturbations, enabling the re-entry reduces the RMSE from 0.19 m to 0.12 m (37% reduction) and increases the in-band percentage from 29.4% to 51.4%.

Overall, the tested configurations demonstrate predictable trends in the controller response, which can be used as practical guidelines for parameter tuning in real deployments.

### 4.4. Computational Requirements and Real-Time Feasibility

A real-time feasibility analysis was conducted using the best-performing configuration (ID2: R=[1.0,2.0], $L_{\mathrm{smooth}}=0.25$ m, cubic interpolation), both with and without the re-entry strategy. The controller was implemented in Python 3.8 using CVXPY as the modeling framework and OSQP as the numerical QP solver [20,21], and executed on an Intel NUC equipped with an Intel Core i7 at 3.0 GHz and 16 GB RAM.

Table 5 reports the computation times of the complete MPC loop. The average total computation time (Avg. Total) corresponds to the mean duration of one control iteration, including QP formulation, solver execution, reference update and input/output operations. The maximum observed computation time (Max. Total) reflects the worst-case duration measured during execution. The average QP solve time (Avg. QP Solve) represents the effort associated exclusively with solving the quadratic program.

Since the sampling period is 20 ms, the computation time per iteration must remain below this threshold to guarantee real-time execution. The MPC loop satisfies this requirement on average in both configurations, and the QP solve time remains significantly below the sampling period (approximately 12 ms), which confirms that the computational bottleneck is not the MPC optimization problem itself.

Enabling the re-entry strategy increases the average computation time by approximately 2–3 ms, which represents an overhead of 11–15% with respect to the baseline. The maximum observed computation time remains almost unchanged (27.4 ms vs. 27.9 ms). This small increment is mainly due to reference update and feasibility checks, and does not modify the complexity of the MPC problem, since the QP solve time remains essentially the same in both cases (11.8–12.5 ms).

These results demonstrate that the proposed re-entry strategy introduces only a minor computational overhead while providing improved tracking and robustness. Furthermore, the implementation is based on Python 3.8 and ROS2 Foxy Fitzroy, which inherently introduce software overhead. A compiled implementation (for example, C++ with OSQP or an embedded QP solver) would further reduce execution time and provide additional margin. Overall, the proposed controller is compatible with real-time execution under the given 20 ms sampling period, even when the re-entry strategy is active.

## 5. Discussion

The experimental results demonstrate the effectiveness of the proposed contributions: the real-time implementation of MPC for trajectory tracking in a DDMR under ROS 2, the novel dynamic look-ahead re-entry strategy for band exits, and the robustness gains confirmed by systematic perturbation trials. Under nominal conditions, the best performance was obtained in configuration ID 2, where the re-entry strategy was active with R=[1.0,2.0], smoothing distance $L_{\mathrm{smooth}}=0.25$ m, and cubic interpolation. This setting yielded the lowest lateral error (RMSE 0.05 m, $P_{95}=0.10$ m) and the highest in-band ratio (68.8%). When perturbations were introduced, the same configuration (ID 6) achieved the most stable recovery, maintaining RMSE of 0.12 m and more than 51% of the trajectory within the band, while weaker penalization (ID 7) or the absence of re-entry (ID 8) led to substantial error growth and longer excursions. These observations confirm that the re-entry trajectory smooths deviations without saturating the control inputs, fulfilling the second contribution of this work.

The literature also highlights the importance of carefully tuning the horizon and weightings in MPC to prevent oscillations or excessive delays. Xu et al. [1] reported improved heading and lateral accuracy in a 3WID3WIS robot by using dynamic MPC, especially in curved paths, which is consistent with our finding that interpolation and smoothing distance strongly affect the correction dynamics. Similarly, Bouzoualegh et al. [6] compared kinematic and dynamic MPC for DDMRs, showing that more realistic models can improve accuracy but increase sensitivity to disturbances; in our case, the re-entry strategy mitigated this sensitivity by reshaping the reference rather than relying solely on the internal model. Abed et al. [3] and Pang et al. [4] introduced fractional and time-varying MPC approaches that enhance stability and robustness, while Ali et al. [2] embedded safety guarantees through barrier functions. By contrast, our approach addresses robustness at the trajectory level; when the robot leaves the nominal path, a smooth local reference is reconstructed, guiding the system back with moderated curvature. This complements but does not replace robust/tube MPC formulations [10,22], which tighten constraints around the nominal trajectory, or learning-based MPC [5,13], which rely on data to compensate for uncertainties.

Recent work in dynamic trajectory re-planning further reinforces the importance of adapting the reference rather than depending exclusively on the predictive model. Lei et al. [15] and She et al. [17] demonstrated that online replanning improves performance in uncertain environments by modifying the trajectory when disturbances or dynamic changes appear. Likewise, Zhang et al. [16] showed that dynamic obstacle avoidance benefits from modifying the local path rather than only tightening constraints. These studies support the motivation behind the re-entry strategy proposed here; providing a lightweight and smooth adjustment of the reference allows rapid recovery from deviations without increasing the MPC complexity or altering the model.

In contrast, the hierarchical framework by Wang et al. [23] integrates MPC with local planners (DWA) and adapts horizon and reference points in real time, achieving improvements in tracking error and reference path following under complex navigation tasks. The re-entry strategy directly reduces the duration and amplitude of error peaks, as evidenced in P1–P3, fulfilling the third contribution of this paper.

If a quantitative comparison is made with related works, it should be considered that the reported performance metrics depend strongly on each robot’s mechanical design, sensor configuration, and testing environment. Therefore, quantitative comparisons are provided only to offer a general reference framework rather than a direct benchmark. For example, recent studies on skid-steer robots using NMPC achieved lateral RMSEs around 0.10–0.15 m in outdoor conditions [22], while we reached 0.05 m under nominal conditions and 0.12 m under perturbations. Gao et al. [9] reported robust tube MPC with cross-track errors between 0.08 and 0.12 m, values comparable to our perturbed cases but obtained without an explicit re-entry mechanism.

Other quantitative benchmarks from recent work further contextualize the improvements observed here. Wang et al. [13] showed that an NMPC enhanced with Gaussian processes reduced lateral RMSE by approximately 15.1% compared to a linear MPC with simple feedback under nominal conditions. This level of improvement is consistent with our configuration ID 2 (lateral RMSE 0.05 m), which exhibited similarly reduced tracking errors relative to less regularized configurations. Likewise, Chen et al. [10] demonstrated that Tube-RMPC decreased maximum trajectory deviation by 9.17% in S-curve maneuvers and reduced MSE by up to 29.38% in double lane change scenarios. Finally, Ravankar et al. [24] highlighted that arc-length parameterization with smoothing interpolation enhances tracking stability and reduces oscillations in mobile robot controllers—consistent with our observation that cubic interpolation with moderate smoothing distances improves re-entry robustness. Overall, although these results were obtained under different experimental setups, the comparative magnitudes suggest that the proposed re-entry strategy and cubic smoothing achieve tracking performance that is quantitatively competitive and qualitatively robust under both nominal and perturbed conditions.

The best-performing configuration in all eight experiments was ID 2/6, where the MPC controller achieved an effective trade-off between control smoothness and tracking accuracy. Its balanced input penalization, cubic interpolation, and moderate smoothing distance minimized both steady-state and transient errors, as reflected in low rate and jerk magnitudes together with a high smoothness index (ISN≈0.95). In particular, the re-entry strategy mitigated the accumulation of large deviations, shortened recovery times, and promoted smoother control profiles than the baseline MPC without re-entry.

However, some limitations remain and they point to concrete avenues for future research. First, the evaluation was restricted to a single robot platform and a flat outdoor environment; performance may vary on uneven terrain, at higher speeds, or with different sensing conditions. Second, formal safety mechanisms such as barrier functions [2] or invariant tubes [10] were not considered in this work, but they could be integrated to enhance safety guarantees. Third, the re-entry mechanism could be extended toward full dynamic replanning or adaptive tuning of $L_{\mathrm{smooth}}$ based on the measured deviation, or combined with learning-based corrections, as suggested in [13,25].

These directions outline the most promising next steps to improve the generalizability, safety guarantees, and applicability of the proposed method.

## 6. Conclusions

In this study, an MPC controller with a novel dynamic look-ahead re-entry strategy was implemented and validated on a four-wheel differential-drive robot under ROS 2. The proposed approach was experimentally tested along a nominal path under both nominal conditions and external perturbations.

Quantitative results demonstrated that, in nominal runs, the best-performing configuration (ID 2) achieved a lateral RMSE of 0.05 m, a heading RMSE of 0.056 rad, and an in-band ratio of 68.8%, outperforming other tested settings. Under perturbations, the re-entry strategy showed clear benefits; for instance, experiment ID 6 maintained a lateral RMSE of 0.12 m and a heading RMSE of 0.45 rad, while still keeping the robot within the validation band for 51.4% of the path, evidencing improved robustness compared to MPC without re-entry. Overall, these results confirm that the proposed re-entry mechanism with cubic smoothing interpolation enhances trajectory tracking stability and robustness, particularly in the presence of disturbances.

Future work will focus on adaptive tuning of the smoothing horizon $L_{\mathrm{smooth}}$ based on the measured deviation, as well as on combining the re-entry strategy with learning-based corrections to further improve performance in complex environments. This study highlights the potential of the proposed method as a baseline for future control architectures in autonomous mobile robots.

Beyond these contributions, the results of this work naturally lead to several avenues for future research. A first direction is the extension of the re-entry mechanism toward full dynamic trajectory re-planning, where the reference could be reshaped online in response to obstacles or environmental changes. A second direction is the integration of the method with safety-aware MPC formulations (such as control barrier functions or tube-based MPC), which would provide formal guarantees under constraints and uncertainties. A third opportunity lies in the adaptive or learning-based adjustment of the smoothing parameters and interpolation mode, so that the controller can automatically adapt to terrain, speed or disturbance variations. Finally, further investigation in embedded implementations or multi-robot settings would enable a broader range of practical deployments. These directions highlight the potential of the re-entry mechanism as a foundation upon which more comprehensive MPC-based navigation architectures can be developed.

## Figures and Tables

**Table 1:** Experiments without perturbations (IDs 1–5).

ID	Re-Entry	*R*	$L_{\mathrm{smooth}}$ [m]	Interp.	Justification
1	Active	[1.0,2.0]	0.30	Linear	Baseline with re-entry. Tests linear interpolation vs. cubic.
2	Active	[1.0,2.0]	0.25	Cubic	Re-entry with cubic interpolation. Shorter look-ahead to speed re-entry.
3	Active	[0.5,1.0]	0.30	Cubic	Smaller *R* to allow stronger control action. Evaluates aggressiveness of corrections.
4	Active	[2.0,4.0]	0.30	Cubic	Larger *R* to emphasize smoother control commands. Explores accuracy vs. smoothness trade-off.
5	Inactive	[1.0,2.0]	–	–	Baseline without re-entry. Used as comparison against active strategy.

**Table 2:** Experiments with perturbations (IDs 6–8).

ID	Re-Entry	*R*	$L_{\mathrm{smooth}}$ [m]	Interp.	Justification
6	Active	[1.0,2.0]	0.25	Cubic	Re-entry with cubic interpolation. Shorter look-ahead for faster recovery.
7	Active	[0.5,1.0]	0.30	Cubic	Re-entry with reduced *R* values. Tests robustness with stronger control effort.
8	Inactive	[1.0,2.0]	–	–	Baseline without re-entry. Used as reference under perturbations.

**Table 3:** Quantitative results of experiments under nominal conditions (IDs 1–5).

Section	Metric	ID 1 (A,[1,2], 0.30, L)	ID 2 (A,[1,2], 0.25, C)	ID 3 (A,[0.5,1.0], 0.30, C)	ID 4 (A,[2,4], 0.30, C)	ID 5 (I,[1,2], 0.30, C)
Trajectory	RMSE $e_{y}$ [m]	0.06	0.05	0.15	0.10	0.07
	MAE $e_{y}$ [m]	0.04	0.04	0.12	0.07	0.06
	P95 $e_{y}$ [m]	0.12	0.10	0.27	0.18	0.15
	RMSE $e_{\theta }$ [rad]	0.13	0.06	0.34	0.09	0.06
	MAE $e_{\theta }$ [rad]	0.10	0.04	0.27	0.07	0.05
	P95 $e_{\theta }$ [rad]	0.28	0.11	0.64	0.18	0.13
	Pct InBand [%]	63.3	68.8	25.2	48.5	52.6
Controller	RateRMS $\dot{v}$ [m/s^2^]	1.90	1.05	4.24	0.87	1.44
	RateRMS $\dot{\omega }$ [rad/s^2^]	3.70	8.21	16.88	8.12	1.43
	JerkRMS $\ddot{v}$ [m/s^3^]	135.29	68.44	272.10	59.83	101.20
	JerkRMS $\ddot{\omega }$ [rad/s^3^]	225.50	661.33	1310.98	655.11	88.94
	ISN [-]	0.96	0.95	0.92	0.94	0.97

**Table 4:** Quantitative results of experiments under perturbations (IDs 6–8).

Section	Metric	ID 6 (A,[1,2], 0.25, C)	ID 7 (A,[0.5,1.0], 0.30, C)	ID 8 (I,[1,2], 0.30, C)
Trajectory	RMSE $e_{y}$ [m]	0.12	0.19	0.19
	MAE $e_{y}$ [m]	0.08	0.15	0.13
	P95 $e_{y}$ [m]	0.25	0.37	0.37
	RMSE $e_{\theta }$ [rad]	0.45	0.46	0.47
	MAE $e_{\theta }$ [rad]	0.24	0.34	0.22
	P95 $e_{\theta }$ [rad]	1.16	0.85	1.28
	Pct InBand [%]	51.4	27.0	29.4
Controller	RateRMS $\dot{v}$ [m/s^2^]	2.19	4.09	3.50
	RateRMS $\dot{\omega }$ [rad/s^2^]	10.34	14.20	5.46
	JerkRMS $\ddot{v}$ [m/s^3^]	146.08	257.53	236.41
	JerkRMS $\ddot{\omega }$ [rad/s^3^]	773.85	1058.09	375.24
	ISN [-]	0.95	0.94	0.97

**Table 5:** Computation time statistics for the MPC loop.

Mode	Avg. Total [ms]	Max. Total [ms]	Avg. QP Solve [ms]
MPC without re-entry	17.2–18.0	27.9	11.8–12.1
MPC with re-entry	19.8–21.6	27.4	12.2–12.5

## Data Availability

Dataset available on request from the authors.
